# Intravascular Brachytherapy for In-Stent Restenosis in Patients With Chronic Kidney Disease

**DOI:** 10.1016/j.jscai.2025.103877

**Published:** 2025-09-09

**Authors:** Gal Sella, Chloe Kharsa, Mangesh Kritya, Devin Olek, Bin S. Teh, Muhammad Faraz Anwaar, Joseph Elias, Elia El Hajj, Albert E. Raizner, Andrew Farach, Neal S. Kleiman, Alpesh Shah

**Affiliations:** a The Heart Center, Kaplan Medical Center, Rehovot, affiliated with the Hebrew University of Jerusalem, Israel; b Department of Cardiology, Houston Methodist DeBakey Heart and Vascular Center, Houston, Texas; c Department of Radiation Oncology, Houston Methodist Hospital, Houston, Texas

**Keywords:** chronic kidney disease, coronary intervention, in-stent restenosis, intravascular brachytherapy, target lesion revascularization, vascular brachytherapy

## Abstract

**Background:**

In-stent restenosis (ISR) remains a significant challenge in coronary intervention, particularly among patients with chronic kidney disease (CKD) who exhibit altered inflammatory responses and accelerated vascular calcification. Vascular brachytherapy has reemerged as a potential treatment modality for recurrent ISR. This study evaluates the clinical outcomes of vascular brachytherapy for ISR in patients with and without CKD.

**Methods:**

We conducted a retrospective analysis of 227 patients (54 in CKD and 173 in non-CKD groups) who underwent vascular brachytherapy for coronary ISR between June 2016 and January 2024 at the Houston Methodist Hospital. Patients were stratified based on the presence of CKD, defined as an estimated glomerular filtration rate of <60 mL/min/1.73 m^2^ for at least 3 months. The primary end point was target lesion revascularization (TLR). Secondary end points included major adverse cardiovascular events, its components and all-cause mortality at 1 year.

**Results:**

Baseline demographic characteristics and procedural characteristics were similar between groups, except for a significantly higher prevalence of diabetes in the CKD group (83.3% vs 57.8%; *P* = .001). At 1-year follow-up, major adverse cardiovascular events (MACE) rates were significantly higher in patients with CKD than those in patients without CKD (63.0% vs 32.9%; *P* = .0003), primarily driven by higher TLR rates (31.5% vs 17.9%; *P* = .038). Bleeding complications occurred exclusively in the CKD group (5.6% vs 0%; *P* = .013). In multivariable analysis, male sex was associated with a significantly lower risk of TLR in patients with CKD (hazard ratio, 0.15; 95% CI, 0.04-0.64; *P* = .010).

**Conclusions:**

Despite similar procedural characteristics, patients with CKD experience significantly higher rates of adverse events following vascular brachytherapy for ISR than those without CKD patients. The nearly doubled rate of MACE and higher TLR rates suggest that the inflammatory milieu associated with CKD may overcome the antiproliferative effects of radiation therapy. These findings highlight the need for refined interventional strategies and comprehensive cardiovascular risk management in this high-risk population with MACE.

## Introduction

In-stent restenosis (ISR) represents a significant challenge in the management of cardiovascular disease, particularly among patients with chronic kidney disease (CKD) who face an elevated risk of this complication.^1^ Brachytherapy emerged as a targeted therapeutic approach for treating ISR in the late 1990s. This treatment modality involves the temporary placement of radioactive sources directly at the site of restenosis, allowing for the delivery of precise, localized radiation.^2^

Clinical evidence regarding brachytherapy in patients with CKD was very limited over the past 2 decades. Early studies demonstrated promising results in reducing restenosis rates compared with conventional balloon angioplasty alone.^3^ However, the widespread adoption of drug-eluting stents led to a decline in brachytherapy use.

The unique vascular biology of patients with CKD influences both the efficacy and potential complications of brachytherapy. Their tendency toward accelerated arterial calcification and altered wound healing responses may necessitate careful consideration of radiation doses and treatment planning.^4^^,^^5^

We aimed to explore the 1-year outcomes of brachytherapy for the treatment of ISR among patients with chronic kidney disease. To the best of our knowledge, no similar study has been published in the existing literature.

## Materials and methods

### Study population and data collection

We conducted a retrospective analysis using data from the Houston Methodist Hospital Brachytherapy Registry, which represents one of the largest single-center collections of vascular brachytherapy procedures. The registry includes 227 patients who underwent vascular brachytherapy for ISR between June 2016 and January 2024, with a minimum follow-up period of 1 year. All procedures were performed at our tertiary care center using standardized protocols.

Data collection used established registry protocols with comprehensive databases capturing detailed patient information, including baseline demographic characteristics, cardiovascular risk factors, clinical presentation, a cardiac history, and complete procedural characteristics. Postprocedural follow-up used comprehensive medical record review supplemented by clinical encounters. Repeat coronary angiography was performed when clinically indicated, such as in cases of recurrent symptoms or objective evidence of ischemia. The follow-up data collection process included documentation of all major adverse cardiac events, with particular attention to target lesion revascularization (TLR) procedures, myocardial infarctions (MIs) and cardiovascular-related hospitalizations. If data were missing, phone calls were permitted to obtain the missing information.

### Patient classification

Patients were stratified into 2 groups based on their renal function: those with CKD group and those with normal renal function (control group). The CKD was defined according to the Kidney Disease: Improving Global Outcomes criteria as an estimated glomerular filtration rate of <60 mL/min/1.73 m^2^ for at least 3 months.^6^ The estimated glomerular filtration rate was calculated using the CKD-EPI equation.^7^

### Clinical event adjudication

Target vessel MI was defined per the Universal Definition of MI, requiring biomarker elevation with clinical or electrocardiographic evidence of ischemia attributable to the target vessel.^8^^,^^9^ Stent thrombosis was classified according to Academic Research Consortium definitions as definite or probable based on clinical presentation and angiographic findings.^10^ All events were adjudicated by 2 independent adjudicators (G.S., C.K.) blinded to renal function status; disagreements were resolved by consensus.

### Brachytherapy procedure

Procedures were performed according to the vendor recommended protocol and after neointimal proliferation process was suspected to be involved in the ISR mechanism per intravascular ultrasound (IVUS) imaging (when used). After successful balloon angioplasty of the restenotic lesion, β radiation was delivered using a 7F guide catheter with the Beta-Cath 3.5F System (Novoste Corporation). The source train comprised strontium/ytrium-90 seeds. To ensure complete lesion coverage and account for edge effects, the radiation source train length was selected to exceed the angioplasty segment by 10.0 mm on each end. Due to guide catheter placement issues or specific patient conditions, reduced margins were accepted in some cases. Radiation dosing followed a vessel size-dependent protocol, with all doses prescribed at 2.0 mm from the radioactive source center: vessels diameters of ≤3.25 mm received 18.4 Gy, while vessel diameters of >3.25 mm received 23 Gy. Long lesions that required multiple contiguous dwells had an overlap segment of approximately 10.0 mm.

### Antithrombotic regimen

All patients received periprocedural anticoagulation with either intravenous heparin (70 IU/kg or 5000 IU) or bivalirudin (0.75 mg/kg bolus followed by 1.75 mg/kg/h infusion). Additional doses were administered as needed to maintain therapeutic anticoagulation. The postprocedural antithrombotic regimen included clopidogrel (75.0 mg/d following a 300.0- to 600.0-mg loading dose) for 12 months and lifelong aspirin (75.0-100.0 mg/d) as part of our institutional policy. Prasugrel or ticagrelor could be substituted for clopidogrel based on guidelines adopted at time of the procedure and clinical considerations.^11^, ^12^, ^13^ Preloading with any of these medications was permitted.

### Study end points

Baseline demographic characteristic data, cardiovascular risk factors, medication use, and procedural characteristics were collected from electronic medical records. Angiographic parameters, including lesion length and reference vessel diameter, were visually estimated by certified experienced interventional cardiologists. The primary end point was TLR at 1 year. Secondary end points included 1-year mortality and MACE defined as a composite of cardiac death, target vessel MI, TLR, stent thrombosis, and bleeding events as defined by the Bleeding Academic Research Consortium criteria.^10^ We included bleeding in the major adverse cardiovascular events (MACE) definition due to the prolonged duration of dual antiplatelet therapy (12 months).

### Statistical analysis

Continuous variables were expressed as mean ± SD or median (IQR), as appropriate. Categorical variables were presented as frequencies and percentages. Comparisons between CKD and non-CKD groups were performed using Student *t* test or Mann-Whitney *U* test for continuous variables and χ^2^ or Fisher exact test for categorical variables. Time-to-event analyses were conducted using Kaplan-Meier methods, with group differences assessed by the log-rank test.

Cox proportional hazards models were used to identify independent predictors of adverse events. Candidate variables for multivariable analysis were selected based on clinical relevance and included age, sex, diabetes mellitus, left ventricular ejection fraction (EF) of <50%, body surface area, and CKD status. Hazard ratios (HRs) with corresponding 95% CIs were reported. All statistical analyses were performed using Python version 3.12.7, with a 2-sided *P* value of <.05 considered statistically significant.

### Study oversight

The study protocol was approved by the Institutional Review Board of Houston Methodist Hospital (PRO00025173). Written informed consent was waived for all patients because it is a retrospective chart review study. The study was conducted in accordance with the Declaration of Helsinki and Good Clinical Practice guidelines.

## Results

### Baseline patient characteristics

A total of 227 patients were included in the analysis, with 54 patients (23.8%) classified into the CKD group and 173 patients (76.2%) into the non-CKD group. Baseline demographic characteristics were similar between the groups. Mean age was 66.6 ± 11.1 years in the CKD group vs 65.0 ± 10.5 years in the non-CKD group (*P* = .34). The proportion of male patients was 66.7% vs 73.4% (*P* = .43), and body surface area was 1.97 ± 0.26 vs 2.03 ± 0.31 m^2^, respectively (*P* = .19).

Comorbidities included diabetes mellitus (83.3% vs 57.8%; *P* = .001), hypertension (100% vs 95.4%; *P* = .20), hyperlipidemia (90.7% vs 92.5%; *P* = .90), reduced left ventricular EF (<50%; 25.9% vs 16.2%; *P* = .16), chronic obstructive pulmonary disease (14.8% vs 6.4%; *P* = .09), and active smoking (38.9% vs 29.7%; *P* = .27) (Table 1).

**Table 1:** Baseline patient characteristics.

Variable	CKD (n = 54)	Non-CKD (n = 173)	*P*
Age, y	66.6 ± 11.1	65.0 ± 10.5	.34
Male sex	36 (66.7)	127 (73.4)	.43
Body surface area, m^2^	1.97 ± 0.26	2.03 ± 0.31	.19
EF <50%	14 (25.9)	28 (16.2)	.16
COPD	8 (14.8)	11 (6.4)	.09
Hypertension	54 (100)	165 (95.4)	.20
Hyperlipidemia	49 (90.7)	160 (92.5)	.90
Diabetes mellitus	45 (83.3)	100 (57.8)	.001
Active smoker	21 (38.9)	51 (29.7)	.27

Values are n (%) or mean ± SD.

CKD, chronic kidney disease; COPD, chronic obstructive pulmonary disorder; EF, ejection fraction.

### Procedural characteristics

Procedural characteristics were comparable between the 2 groups. Procedure duration (91.1 ± 43.7 vs 89.6 ± 40.1 minutes; *P* = .89), contrast volume (149.8 ± 76.7 vs 144.2 ± 58.5 mL; *P* = .95), and reference vessel size (3.58 ± 0.56 vs 3.51 ± 0.64 mm; *P* = .39) were not different. Lesion length was 26.8 ± 16.3 mm in patients with CKD and 30.5 ± 23.3 mm in those without CKD (*P* = .19). Stent implantation was performed in 18.5% of patients with CKD and 17.3% of those without CKD (*P* = .84). IVUS guidance was used in 68.5% of patients with CKD and 60.1% of those without CKD (*P* = .34).

Plaque modification strategies, including laser atherectomy (16.7% vs 19.7%; *P* = .69) and intravascular lithotripsy (13.0% vs 4.6%; *P* = .05), were used with similar frequency between groups. Acute procedural complications, including dissection, perforation, and acute thrombosis, were rare and comparable across groups (Table 2).

**Table 2:** Procedural characteristics.

Variable	CKD (n = 54)	Non-CKD (n = 173)	*P*
Procedure length, min	91.1 ± 443.7	89.6 ± 40.1	.89
Radiation dose, mGy/cm^2^	18,195 ± 17,119	24,836 ± 24,716	.10
Contrast volume, mL	149.8 ± 76.7	144.2 ± 58.5	.95
Reference vessel size, mm	3.58 ± 0.56	3.51 ± 0.64	.39
Lesion length, mm	26.8 ± 16.3	30.5 ± 23.3	.19
Vessel size, <2.75 mm	4 (7.4)	16 (9.2)	.89
Stent implantation during procedure	10 (18.5)	30 (17.3)	.84
Two dwells	13 (24.1)	35 (20.2)	.57
Intravascular ultrasound	37 (68.5)	104 (60.1)	.34
Artery			
Left anterior descending	16 (29.6)	56 (32.4)	.83
Left circumflex	19 (35.2)	39 (22.5)	.09
Right	12 (22.2)	50 (28.9)	.43
Ramus intermedius	1 (1.9)	7 (4.0)	.73
Left main coronary artery	4 (7.4)	11 (6.4)	>.99
Balloon preparation type			
Semicompliant balloon	3 (5.6)	14 (8.1)	.75
Noncompliant balloon	25 (46.3)	98 (56.6)	.24
Scoring balloon	4 (7.4)	5 (2.9)	.28
Cutting balloon	18 (33.3)	52 (30.1)	.78
Plaque modification			
Laser	9 (16.7)	34 (19.7)	.69
Intravascular lithotripsy	7 (13.0)	8 (4.6)	.053
Acute complications			
Severe dissection	0 (0)	1 (0.6)	>.99
Acute thrombosis	1 (1.9)	0 (0)	.24
Perforation	1 (1.9)	3 (1.7)	>.99

Values are n (%) or mean ± SD.

CKD, chronic kidney disease.

### One-year clinical outcomes

At 1-year follow-up, MACE occurred in 63.0% of patients in the CKD group compared with 32.9% in the non-CKD group (*P* = .0003). TLLR was performed in 31.5% of patients in the CKD group compared with 17.9% of in the non-CKD group (*P* = .038). MI occurred in 11.1% vs 6.4% (*P* = .25), cardiac hospitalizations in 33.3% vs 23.1% (*P* = .15), and cardiac death in 3.7% vs 0.6% (*P* = .14), respectively. All-cause mortality was 7.4% in the CKD group compared with 4.0% in the non-CKD group (*P* = .30). Bleeding events occurred only in the CKD group (5.6% vs 0%; *P* = .013). Stent thrombosis rates were low and similar (1.9% vs 1.2%; *P* = .56) (Table 3; Central Illustration).

**Table 3:** One-year outcomes.

Outcome	CKD (n = 54)	Non-CKD (n = 173)	*P*
MACE	34 (63.0)	57 (32.9)	.0003
TLR	17 (31.5)	31 (17.9)	.038
Thrombosis	1 (1.9)	2 (1.2)	.56
Bleeding	3 (5.6)	0 (0)	.013
Cardiac hospitalizations	18 (33.3)	40 (23.1)	.15
MI	6 (11.1)	11 (6.4)	.25
Cardiac death	2 (3.7)	1 (0.6)	.14
All-cause mortality	4 (7.4)	7 (4.0)	.30

Values are n (%).

CKD, chronic kidney disease; MACE, major adverse cardiac event; MI, myocardial infarction; TLR, target lesion revascularization.

**Central Illustration:**
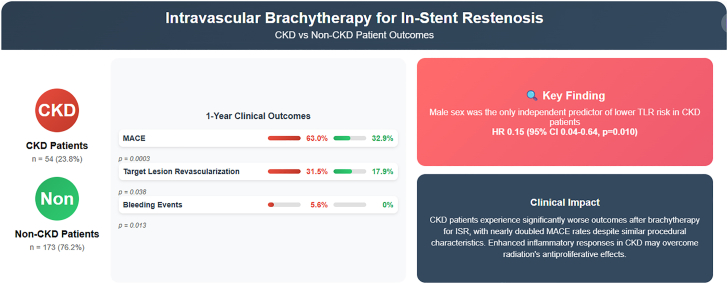
**Clinical outcomes following vascular brachytherapy forin-stentrestenosis in patients with and without chronic kidney disease (CKD).** This schematic summarizes the comparative 1-year rates of major adverse cardiovascular events (MACE), target lesion revascularization (TLR), and bleeding events. Patients with CKD experienced significantly worse outcomes across all end points, highlighting the prognostic impact of renal dysfunction in this high-risk population.

### Kaplan-Meier survival analysis

**Figure 1:**
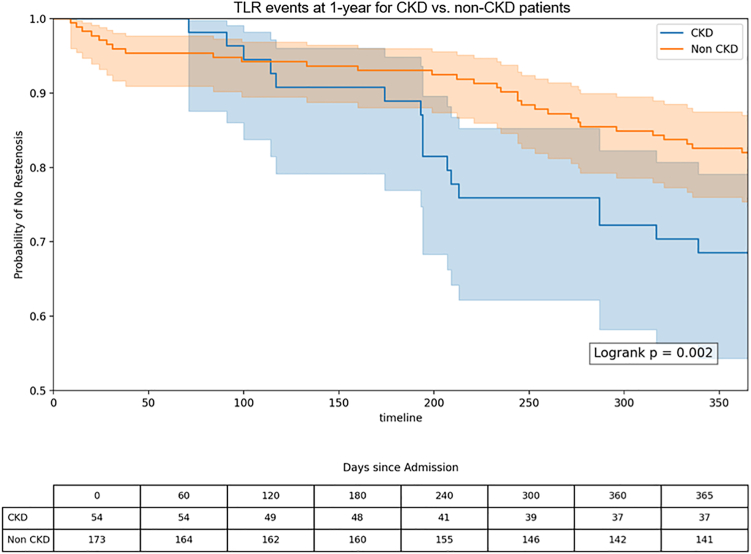
**Kaplan-Meier curves for 1-year target lesion revascularization (TLR) in patients with and without chronic kidney disease (CKD) undergoing vascular brachytherapy for in-stent restenosis.** Patients with CKD exhibited a higher cumulative incidence of TLR than those without CKD. The log-rank test was used to assess differences between groups.

**Figure 2:**
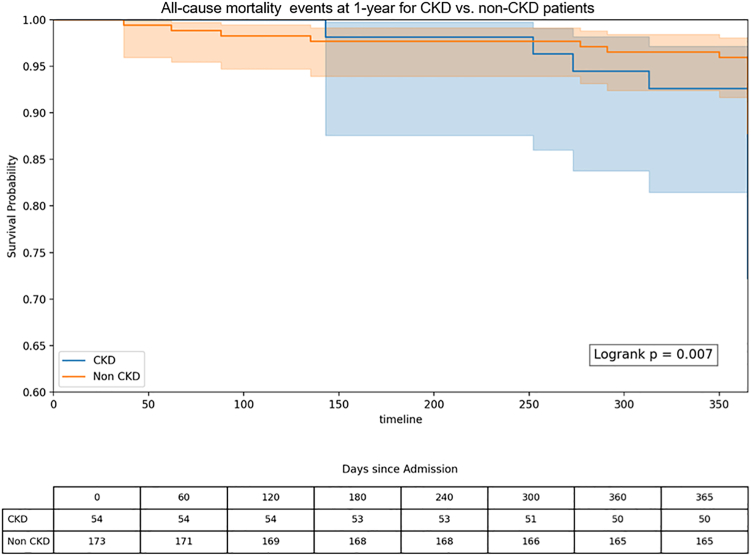
**Kaplan-Meier curves for 1-year all-cause mortality in patients with and without chronic kidney disease (CKD) undergoing vascular brachytherapy for in-stent restenosis.** Patients with CKD experienced significantly higher mortality rates than those without CKD. The log-rank test was used to assess differences between groups.

Kaplan-Meier curves demonstrated a significantly lower event-free survival in the CKD group than that in the non-CKD group at 1 year. Patients with CKD experienced earlier and more frequent occurrence of TLR throughout follow-up (log-rank *P* = .002) (Figure 1). Overall survival was significantly different between groups, with higher incidence of all-cause mortality at 1 year for the CKD group (log-rank *P* = .007) (Figure 2).

### Predictors of TLR in patients with CKD

**Figure 3:**
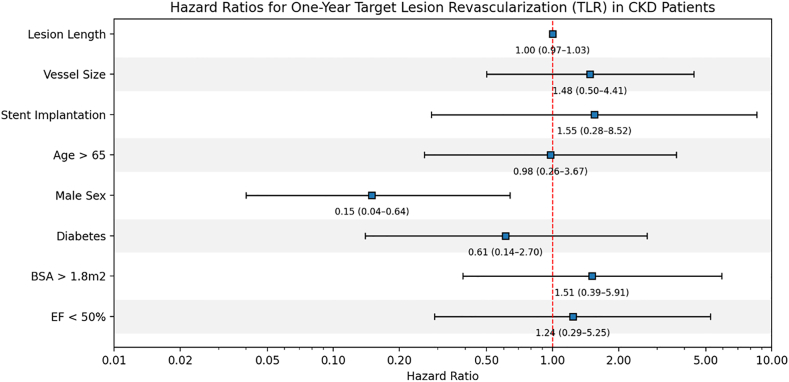
**Forest plot of hazard ratiosfor 1-year target lesion revascularization (TLR) following vascular brachytherapy for in-stent restenosis.** The plot summarizes univariable and multivariable Cox proportional hazards models assessing the association between baseline clinical and angiographic characteristics and the risk of TLR. Error bars represent 95% CIs. BSA, body surface area; CKD, chronic kidney disease; EF, ejection fraction.

In multivariable analysis limited to the CKD cohort, male sex was independently associated with a significantly lower risk of TLR (HR, 0.15; 95% CI, 0.04-0.64; *P* = .010). Lesion length (HR, 1.00; 95% CI, 0.97-1.03; *P* = .76), vessel size (HR, 1.48; 95% CI, 0.50-4.41; *P* = .48), stent implantation (HR, 1.55; 95% CI, 0.28-8.52; *P* = .61), age > 65 years (HR, 0.98; 95% CI, 0.26-3.67; *P* = .98), presence of diabetes (HR, 0.61; 95% CI, 0.14-2.70; *P* = .52), body surface area > 1.8 m^2^ (HR, 1.51; 95% CI, 0.39-5.91; *P* = .56), and EF < 50% (HR, 1.24; 95% CI, 0.29-5.25; *P* = .77) were not independently associated with TLR risk (Table 4; Figure 3).

**Table 4:** Hazard ratio of 1-year target lesion revascularization for patients with chronic kidney disease.

Variable	HR (95% CI)	*P*
Lesion length	1.0 (0.97-1.03)	.76
Vessel size	1.48 (0.5-4.41)	.48
Stent implantation	1.55 (0.28-8.52)	.61
Age >65 y	0.98 (0.26-3.67)	.98
Male sex	0.15 (0.04-0.64)	.010
Diabetes	0.61 (0.14-2.7)	.52
BSA >1.8 m^2^	1.51 (0.39-5.91)	.56
EF <50%	1.24 (0.29-5.25)	.77

BSA, body surface area; CKD, chronic kidney disease; EF, ejection fraction; HR, hazard ratio; TLR, target lesion revascularization.

## Discussion

Our analysis of the Houston Methodist Hospital Brachytherapy Registry revealed significant differences in outcomes between patients with CKD and those with normal renal function. These findings confirm the historical observation that patients with CKD experience worse cardiovascular outcomes following interventional procedures, despite the theoretical advantages of brachytherapy in this population.^14^^,^^15^

The higher rates of adverse events observed in patients with CKD, particularly the nearly doubled rate of MACE at 1 year (63.0% vs 32.9%), underscore the profound impact of renal dysfunction on cardiovascular outcomes. The major component that contributed most to MACE was TLR, with rates of 31.5% vs 17.9% (*P* = .038). Oliver et al^16^ reported similar intermediate-term (6-24 months) TLR rates to those of our non-CKD group (19.8%). This disparity persisted despite similar procedural characteristics, suggesting that underlying pathophysiological processes associated with CKD, rather than technical aspects of the procedure, are key drivers of these differences. Several mechanisms may explain these findings, including persistent uremia-associated inflammation, accelerated neointimal proliferation, and vascular calcification, which may attenuate the efficacy of intracoronary radiation.

Importantly, the CKD cohort demonstrated a high burden of additional comorbidities, including diabetes mellitus (83%) and reduced left ventricular EF (26%), both of which are recognized contributors to adverse cardiovascular events. Although these factors were not independently associated with TLR in our multivariable analysis, their presence likely contributed to the higher observed MACE rates in the CKD group. The interaction between CKD, metabolic dysfunction, and impaired ventricular function may synergistically promote endothelial dysfunction, systemic inflammation and adverse vascular remodeling, further compounding cardiovascular risk. Given the relatively small sample size within the CKD subgroup, these results should be interpreted with caution, and residual confounding cannot be entirely excluded despite adjustment for key clinical variables.

Our study also identified male sex as the only independent predictor associated with lower TLR risk in patients with CKD (HR, 0.15; 95% CI, 0.04-0.64; *P* = .010). The potential protective effect of male sex in this population is intriguing and warrants further investigation, as it may reflect sex-specific differences in vascular biology, hormonal influence, or healing responses in the context of renal dysfunction.

These findings may have important clinical implications. While CKD should not be viewed as a contraindication to brachytherapy, careful patient selection and individualized risk assessment remain critical. For patients with CKD with recurrent ISR, brachytherapy remains a technically feasible option, especially when alternative strategies are limited due to anatomical constraints or contraindications to prolonged dual antiplatelet therapy. Nevertheless, the persistent high rates of adverse events emphasize the need for continued optimization of adjunctive pharmacotherapy, aggressive risk factor modification, and close long-term follow-up in this vulnerable population.

Our results open several avenues for future research. Further mechanistic studies exploring the complex interplay between renal dysfunction, metabolic disease, and vascular response to brachytherapy may help refine treatment strategies. Additionally, larger prospective studies with longer-term follow-up are needed to validate our findings and to better define the role of brachytherapy in the contemporary management of ISR in patients with CKD.

### Limitations

Our study has several important limitations that should be acknowledged. First, as a single-center retrospective analysis, our findings may reflect institutional expertise and practice patterns specific to our tertiary care center, potentially limiting generalizability to other clinical settings. The nonrandomized nature of our study introduces the possibility of selection bias, despite our efforts to control for known confounders through multivariate analysis.

Second, the relatively small sample size in the CKD group (n = 54) may have limited our ability to detect more subtle differences in outcomes or to identify additional independent predictors of adverse events. The sample size also precluded meaningful subgroup analyses based on CKD severity, which might have revealed differences in outcomes across the spectrum of renal dysfunction.

Third, our study did not include a comparison group of patients with CKD treated with alternative modalities for ISR, such as drug-coated balloons or newer-generation drug-eluting stents. Such comparisons would be valuable in determining the relative efficacy of brachytherapy in this high-risk population.

Fourth, the heterogeneity of the coronary lesion characteristics and an interventional history in our study population may have influenced outcomes independent of renal function, although some recent studies suggest that multiple stent layers are not predictive for 1-year TLR.^17^^,^^18^ While we attempted to account for these factors, unmeasured variables related to lesion complexity and vascular biology may have confounded our results.

Fifth, our follow-up period of 1 year, while providing important insights into medium-term outcomes, may not capture later adverse events that could influence the overall risk-benefit assessment of brachytherapy in patients with CKD. Longer-term follow-up will be necessary to determine the durability of treatment effects and to identify any late complications.

Sixth, while IVUS was used in the majority of cases (∼68% in patients with CKD), systematic characterization of the underlying mechanism of ISR (eg, stent underexpansion, neointimal hyperplasia, or multiple stent layers) could not be uniformly performed. This limits our ability to fully assess lesion-specific contributors to restenosis across the entire cohort.

Finally, our study did not include comprehensive data on medication adherence and lifestyle modifications, which may be particularly relevant in patients with CKD who often have multiple comorbidities requiring complex medical regimens. These factors could have influenced our observed outcomes independent of the brachytherapy procedure itself.

## Conclusion

Our analysis shows that patients with CKD experience significantly higher rates of adverse events following vascular brachytherapy for ISR than those with normal renal function. The nearly 2-fold increase in 1-year MACE underscores the impact of renal dysfunction, despite comparable baseline and procedural characteristics. Elevated TLR rates in patients with CKD may reflect an exaggerated inflammatory and proliferative response that diminishes the efficacy of radiation therapy. Moreover, the occurrence of all bleeding complications exclusively in the CKD group highlights the importance of individualized anticoagulation strategies in this high-risk population.

Despite these challenges, vascular brachytherapy remains a technically feasible and relevant option for patients with CKD and recurrent restenosis, particularly when other strategies are limited by anatomy or contraindications to prolonged dual antiplatelet therapy. The observed protective association of male sex against TLR in this subgroup is intriguing and may inform future patient selection. These findings emphasize the need for refined interventional approaches and comprehensive cardiovascular risk management in patients with CKD. Future research should explore the molecular mechanisms driving differential responses to radiation and evaluate protocol modifications that could improve outcomes in this complex population.
